# Entrapment of Autologous von Willebrand Factor on Polystyrene/Poly(methyl methacrylate) Demixed Surfaces

**DOI:** 10.3390/polym9120700

**Published:** 2017-12-13

**Authors:** Joanna Ward, Eimear Dunne, David Bishop, Adrian Boyd, Dermot Kenny, Brian J. Meenan

**Affiliations:** 1 Nanotechnology and Integrated Bioengineering Centre (NIBEC), Ulster University, Jordanstown BT37 0QB, UK; Ward-J12@ulster.ac.uk (J.W.); Bishop-D1@ulster.ac.uk (D.B.); ar.boyd@ulster.ac.uk (A.B.); 2 Royal College of Surgeons in Ireland, 123 St. Stephen’s Green, Dublin 2, Ireland; edunne@rcsi.ie (E.D.); dkenny@rcsi.ie (D.K.)

**Keywords:** von Willebrand Factor (vWF), polystyrene/poly(methylmethacrylate) demixed solutions, spin coating, surface topography, Dynamic Platelet Function Assay (DPFA)

## Abstract

Human platelets play a vital role in haemostasis, pathological bleeding and thrombosis. The haemostatic mechanism is concerned with the control of bleeding from injured blood vessels, whereby platelets interact with the damaged inner vessel wall to form a clot (thrombus) at the site of injury. This adhesion of platelets and their subsequent aggregation is dependent on the presence of the blood protein von Willebrand Factor (vWF). It is proposed here that the entrapment of vWF on a substrate surface offers the opportunity to assess an individual’s platelet function in a clinical diagnostic context. Spin coating from demixed solutions of polystyrene (PS) and poly(methyl methacrylate) (PMMA) onto glass slides has been shown previously to support platelet adhesion but the mechanism by which this interaction occurs, including the role of vWF, is not fully understood. In this work, we report a study of the interaction of platelets in whole blood with surfaces produced by spin coating from a solution of a weight/weight mixture of a 25% PS and 75% PMMA (25PS/75PMMA) in chloroform in the context of the properties required for their use as a Dynamic Platelet Function Assay (DPFA) substrate. Atomic Force Microscopy (AFM) indicates the presence of topographical features on the polymer demixed surfaces in the sub-micron to nanometer range. X-ray Photoelectron Spectroscopy (XPS) analysis confirms that the uppermost surface chemistry of the coatings is solely that of PMMA. The deliberate addition of various amounts of 50 μm diameter PS microspheres to the 25PS/75PMMA system has been shown to maintain the PMMA chemistry, but to significantly change the surface topography and to subsequently effect the scale of the resultant platelet interactions. By blocking specific platelet binding sites, it has been shown that their interaction with these surfaces is a consequence of the entrapment and build-up of vWF from the same whole blood sample.

## 1. Introduction

Platelets play a vital role in haemostasis and thrombosis [1]. Haemostasis is concerned with the arrest of bleeding from injured blood vessels, whereby platelets are recruited by blood proteins to form a thrombus (clot) [2]. This platelet interaction is specifically governed by the presence of von Willebrand Factor (vWF)—a large glycoprotein that is synthesized in megakaryocytes [3]. In normal blood flow, the cells and platelets are subject to wall shear stresses and the circumferential strain that regulate their distribution within the vessel. These forces push platelets towards the inner lumen surface where they can respond to the adsorbed vWF when it is recruited to the site of damage [3,4]. The normally coiled vWF uncoils on adherence to the collagen that is exposed when the vessel endothelium layer is no longer intact, resulting in the availability of receptors for subsequent platelet interactions [4]. Under these conditions, the platelets initially bind, release and roll off the surface until the vWF has attained its final conformation at which point they adhere permanently thereby commencing the formation of a thrombus. Whereas, this a critically important part of the natural processes for the control of bleeding, obviously if any part of the process does not behave as required then there can be adverse consequences [1].

Rapid and accurate testing of platelet function is essential for the diagnosis, monitoring and control of bleeding disorders such as von Willebrand Disease (vWD); a condition that occurs due to a deficiency in the function of an individual’s vWF and that is estimated to occur in approximately 1% of the global population [5,6]. Although there are a range of platelet function tests (PFT) for the diagnosis of conditions such as vWD, they are extremely limited in terms of their suitability to truly represent the in vivo process of haemostasis. The main limitation of note, is a lack of truly physiological factors being taken into consideration due to these measurements normally being made under static or incorrect flow conditions [7,8,9,10,11]. Recent advances in the area of PFT involve the use of a parallel platelet flow chamber that is capable of passing small volumes of blood over a substrate surface under physiologically relevant wall shear force levels [12,13]. The current version of this dynamic PFT system uses an assay surface in which small volumes of whole blood are brought into contact with immobilised endogenous vWF [14,15]. Whereas, this dynamic assay is a significant advance on those that employ static processes, the inclusion of autologous vWF in this device offers a major advantage, particularly if the desire is for the vWF and subsequent platelet interactions to occur in the same blood sample. To this end, provision of a surface that can both directly entrap vWF from flowing blood and subsequently allow its interaction with platelets thereon is required [14,16,17,18].

Previous studies have shown that the platelets present in whole blood will interact (adhere, roll, stick, etc.) with polymers that have specific forms of nanostructured surface feature [19,20]. These polymer surfaces can be created by the spin coating of polystyrene (PS)/poly(methylmethacrylate) (PMMA) demixed solutions onto glass substrates in various weight/weight (*w/w*) ratio blends and concentrations. The potential for such nanoscale features to then directly entrap autologous vWF from whole blood at arterial shear flow rates (1500 s^−1^) in a way that allows for the subsequent platelet interactions to be measured in a one-step process has been investigated. Whereas, these results confirm that the PS/PMMA demixed spin coated surface do indeed entrap vWF, the surfaces are not optimal for use within a parallel plate dynamic assay device. The distribution of the surface features along the flow path is non-uniform and thereby effects the attendant platelet surface coverage.

This paper reports a major refinement of the polymer demixed thin film deposition process by the introduction of 50 μm diameter PS microspheres to create a more regular and reproducible distribution of the surface features of interest. The topography of the resultant surfaces has been characterised by Atomic Force Microscopy (AFM) and the associated chemistry by X-ray Photoelectron Spectroscopy (XPS). A Dynamic Platelet Function Assay (DPFA) device that normally operates with immobilised endogenous vWF substrates has then been used to assess both their ability to entrap autologous vWF under flowing blood at arterial shear (1500 s^−1^) and to allow for the measurement of subsequent platelet interactions thereon.

## 2. Materials and Methods

### 2.1. Sample Preparation

High grade Polystyrene (PS) and Poly(methylmethacrylate) (PMMA) (Sigma Aldrich, Irvine, UK, *M*_W_ = 290,000 amu and *M*_W_ = 350,000 amu, respectively) were used throughout and combined to create the necessary 25%PS/75%PMMA weight-to-weight ratio (referred to herein as 25PS/75PMMA). In order to make a 3% casting solution of this demixed system 0.75 g of PS was mixed with 2.25 g of PMMA and 97 g (63.7 mL) of chloroform (119.38 g/mol, Sigma Aldrich, Irvine, UK) added. The solution was then placed on a vibrating plate in a stoppered flask for 24 h in ensure full PMMA dissolution and mixing. Under these conditions the PS particles are essentially insoluble.

Aliquots of the main casting solution were modified with the addition of 50 μm PS microsphere microspheres (Thermo Scientific, Loughborough, UK). The microspheres were delivered in aqueous solution in 25 mL vials at 3000 microspheres per mL. 5mL of the PS microsphere solution, providing a total of 15,000 microspheres, was placed onto a rigorously clean watch glass and placed in an oven to dry. The microspheres were then re-suspended in 1 mL of chloroform and added immediately to varying amounts of the stock 25PS/75PMMA solution to create the following ratios: 1:2 (+1500 microspheres), 1:4 (+750 microspheres) and 1:8 (+375 microspheres) per mL of casting solution. There was no evidence found for the gross morphology of the PS microspheres having been changed on exposure to the solvent.

### 2.2. Spin Coating of Polymer Demixed Solutions

Glass coverslips (24 mm × 50 mm × 0.15 mm) (Marienfeld-Superior, Lauda-Königshofen, Germany) were placed in a beaker containing 99% isopropanol (IPA) (Sigma Aldrich, Irvine, UK) in an ultrasonic bath (Ultrawave Ltd., Cardiff, UK) for 30 min to remove any surface contamination. They were then removed, rinsed with deionized water and dried with a lint free cloth before being placed in an oven at 70 °C until required. The clean, dry substrates were placed in the vacuum chuck of a SCS G3P-12 spin coating device (PiKem, Wilnecote, UK) and the polymer demixed casting solutions (with and without various numbers of 50 μm PS microspheres) applied drop wise onto the surface to create a fully coherent liquid layer. The spin program was then initiated which comprised ramping the chuck rotation up to 6000 rpm over a period of three minutes. The sample types are hereafter referred to as 25PS/75PMMA; 25PS/75PMMA + 375; 25PS/75PMMA + 750 and 25PS/75PMMA + 1500.

### 2.3. Optical Microscopy

Optical microscopy was employed to examine the distribution of the PS particles within the PMMA after spin coating from the various PS/PMMA demixed surfaces. Images were obtained using a Nikon TS Eclipse 100 phase-contrast microscope, with a Nikon X20 objective (Nikon, Baldock UK).

### 2.4. Atomic Force Microscopy (AFM)

AFM was used to image the topographical features produced on the various spin coated PS/PMMA demixed surfaces at sub-nanometer resolution and to provide surface roughness values and line profile plots. A Veeco Digital Instrumentation Dimension 3100 AFM (Bruker Axs, Coventry, UK) instrument equipped with a silicone tip and operating in tapping mode was used throughout. A scan rate of 0.5 Hz was used to scan an area of 10 µm by 10 µm. The deflection of the tip from the surface, caused by van der Waals forces and the electrostatic attraction/repulsion between atoms on the surface and the tip, was measured in terms of the amplitude change of the tip cantilever and translated into topographical information via a pseudo colour plot of the *xyz* co-ordinates. Surface roughness was recorded as both *R*_a_ and *R*_q_ values. In this regard, *R*_a_ is the arithmetic values of the profile height deviations from the mean line, recorded within the evaluation limits. For the AFM measurements recorded here, it is then the average of the *z*-axis peaks and valleys represented in the images. By comparison, *R*_q_ is the root-mean-square roughness, which for the AFM data represents the deviations of the *z*-axis data from the mean value. Line profiles of the peak-to-peak amplitude were obtained from the AFM image data sets in a lengthwise plot perpendicular to the *xy* scan direction.

### 2.5. X-ray Photoelectron Spectroscopy (XPS)

XPS was used to characterize the chemical composition of the various spin coated 25PS/75PMMA demixed surfaces, with and without 50 μm PS microspheres. XPS was carried out using a Kratos Axis Ultra DLD Spectrometer (Kratos Analytical Ltd., Manchester, UK) operating with aluminum Kα X-rays at an incident energy of 1486.6 eV. Wide energy survey scans (WESS) were acquired at a pass energy of 160 eV followed by high resolution spectra for the carbon C1s and oxygen O1s regions, respectively, at a pass energy of 20 eV. Given the insulating nature of the polymer demixed samples, in situ charge neutralization was applied via a low energy electron gun operating with a filament current of 1.95 A and a charge balance setting of 3.3 V, working in tandem with a magnetic immersion lens. Correction for any residual charging effects was made by setting the main component of the C1s peak to 284.6 eV, the value associated with adventitious carbon.

### 2.6. Dynamic Platelet Function Assay Measurements

Blood from healthy volunteer donors was collected and stained with 1 μM 3-3′-dihexyloxacarbocyanine iodide (DIOC_6_), a lipophilic dye that emits light at 488 nm. A Dynamic Platelet Function Assay (DPFA) parallel plate flow chamber comprising a top plate with a pre-cut flow channel and input/output ports was placed onto the glass slide substrate with spin coated PS/PMMA layer. A self-adhesive gasket is used to seal the two parts of the assembly together to create the discrete flow path within the chamber. The assembled flow chambers were mounted on a Zeiss Axiovert-200 epifluorescence inverted microscope (Zeiss, Oberkochen, Germany). A NEMESYS syringe pump was then used to perfuse the stained whole blood across the flow path. A flow rate (Q) of 75 μL/min was used corresponding to an arterial shear rate (γ) of 1500 s^−1^ which is equivalent to that experienced on the inner lumen of small arteriole vessels. The flow path of the DPFA device was illuminated with an Osram 103-W light source equipped with a fluorescein iosthiocyanate (FITC) filter (Chroma Technology Corp., Rockingham, VT, USA) providing excitation and emission at 490 nm and 528 nm, respectively. The fluorescent images were obtained using a vacuum-cooled (−80 °C) digital iXON EM+ CCD camera (Andor Technology, Belfast, UK) operating with MetaMorph Microscopy and Automation and Image Analysis Software (Molecular Devices Ltd., Wokingham, UK). When the first platelet sticks to the surface, a recording of images at 30 frames per second (fps) for 120 s is attained with a Nikon ×20 objective Lens (field of view 502 × 501 pixels), followed by a time lapse of 1 fps for 120 frames. In all cases, quantitative assessment of the platelet interaction with the surface was based on the final frame (i.e., 120/120) of the image acquisition sequence. Each of these images was imported into the ImageJ software package (NIH, Bethesda, MD, USA) where it was adjusted in terms of brightness and contrast to allow for clear differentiation of the adhered platelets from the background surface features. The files were then imported into the “Volocity” image processing software package (Perkin-Elmer, Llantrisant, UK) and the platelet interaction surface coverage calculated using the “find objects by % intensity” tool. This was carried out with acceptance limits set to 95 (lower limit) and 100 (upper limit). Areas of interaction were measured in respect to the pixels associated with the adhered platelets and the percentage of surface coverage calculated by dividing these values by the total number of pixels present.

In order to further elucidate the mechanism of platelet adherence to the polymer demixed surfaces, inhibition studies were carried out. The platelet GP1b antibody AK2 was added to the labelled blood sample at 20 μg/mL and the assay carried out as described above. In a separate set of experiments, the vWF antibody 5D2 was added to the labelled blood at 25 μg/mL prior to flowing it over the substrate surface.

## 3. Results

**Figure 1:**
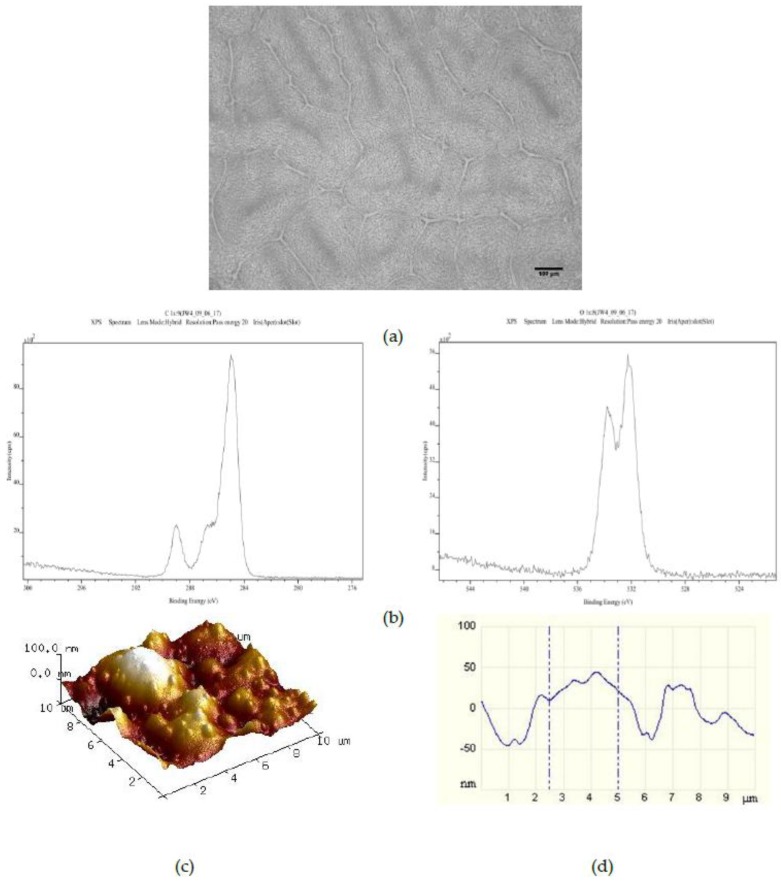
Characterisation of 25PS/75PMMA films by way of (**a**) optical microscopy (×20); (**b**) C1s (**left**) and O1s (**right)** XPS spectra; (**c**) AFM 3D image and (**d**) AFM line profile.

Surfaces created by spin coating from the 25PS/75PMMA solution in chloroform onto glass slides were characterized by optical microscopy, XPS and AFM with typical results shown in Figure 1. The optical imaging (Figure 1a) indicates that the PS is distributed within the PMMA in two distinct arrangements; central circular features and long striations that radiate outwards in various directions. The C1s and O1s regions of the XPS spectra (Figure 1b) confirm that the uppermost surface of the polymer demixed layer is solely that of PMMA with no component features associated with the PS being identified at the quantification limit of the analytical technique (0.1% atomic concentration). The corresponding quantitative data for these C1s and O1s contributions are provided in Table 1, reported as average values (*n* = 3) ± the standard deviation (SD) of measurements of % atomic concentration (%At. Conc.) for each peak from three distinct regions across the surface. The data for a surface created by spin coating pure PMMA in chloroform onto a glass slide is provided for comparison.

The average *R*_a_ and *R*_q_ values (*n* = 3) calculated from the AFM data sets are provided in Table 2. Based on these data, the topography for this spin coated surface can be described as having a combination of poorly defined micron and nano-scale surface features. Specifically, the AFM data indicate that there are pronounced features with full width at half maximum (FWHM) values in the range of 2 to 4 μm overlaid with features with FWHM of 100 nm to 1 µm.

In order to maintain the core chemical characteristics of this demixed system, known to promote platelet adhesion, while promoting the formation of the topographical nanoscale features in preference to the micron size elements, various numbers of 50 μm PS microspheres were added to the 25PS/75PMMA solutions prior to spin coating.

**Figure 2:**
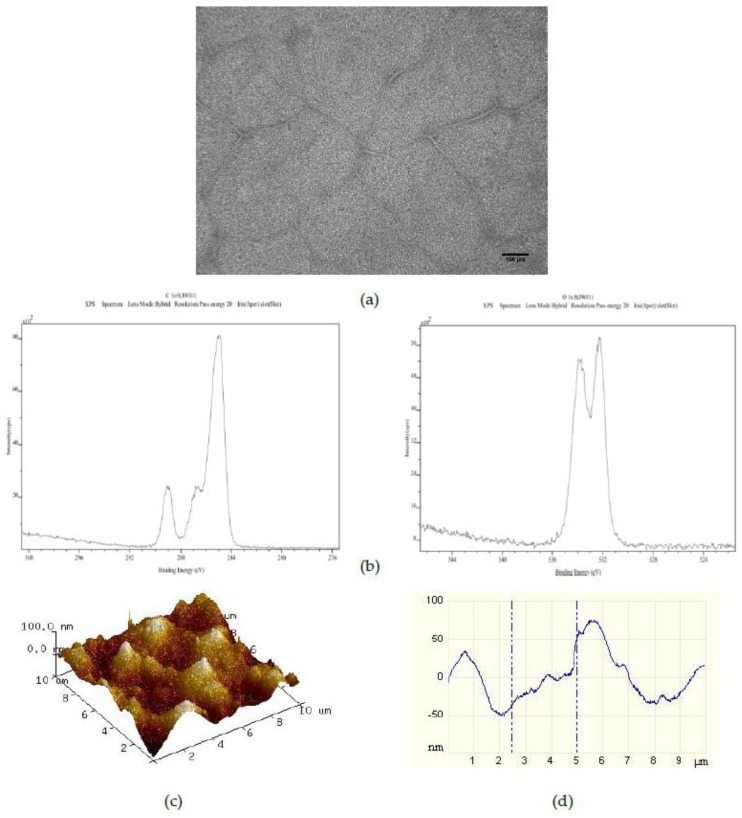
Characterisation of 25PS/75PMMA+375 films by way of (**a**) optical microscopy (×20); (**b**) C1s (**left**) and O1s (**right**) XPS spectra; (**c**) AFM 3D plots and (**d**) AFM line profile.

Addition of 375 PS 50 μm microspheres per mL to the 25PS/75PMMA polymer demixed solution provided a spin coated layer with the surface properties shown in Figure 2. The optical image (Figure 2a) here shows a pattern of striations of the main PS particles which is very similar to that observed for the films from the standard 25PS/75PMMA polymer demixed solution. The C1s and O1s XPS spectra (Figure 2b) are again only that expected for PMMA chemistry. As indicated in Table 1, the relative % atomic concentration C1s and O1s values have not changed. Likewise, the AFM image and line profile for this surface (Figure 2c,d) closely resemble those seen after spin coating from the standard casting solution, with the *R*_a_ and *R*_q_ values also being similar.

**Figure 3:**
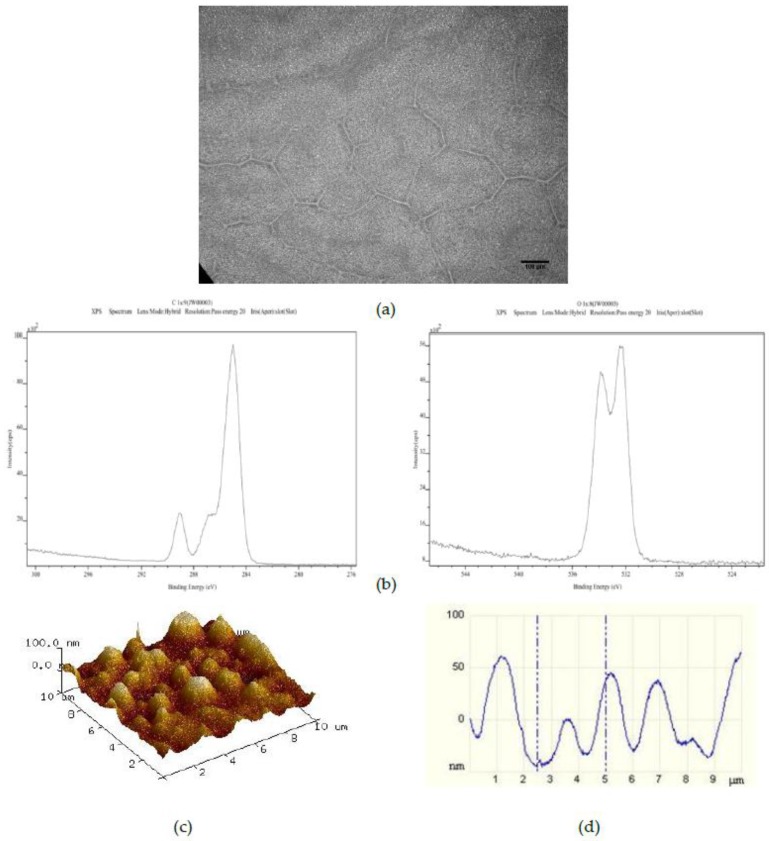
Characterisation of 25PS/75PMMA + 750 films by way of (**a**) optical microscopy (×20); (**b**) C1s (**left**) and O1s (**right**) XPS spectra; (**c**) AFM 3D plots and (**d**) AFM line profile.

The effect of increasing the number of PS 50 μm microspheres added to the standard casting solution to 750 on the resulting spin coated layer is shown in Figure 3. The optical image (Figure 3a) here shows the same randomly distributed striations of PS particles as for the standard and standard plus 375 50 μm PS bead surfaces. The C1s and O1s XPS spectra (Figure 3b) once again indicate a pure PMMA surface chemistry. However, the corresponding AFM image and line profile (Figure 3c,d) indicate the presence of a more highly ordered distribution of recurring surface features in the 40 nm to 50 nm height range with full width at half maximum (FWHM) values of approximately 1 μm.

**Figure 4:**
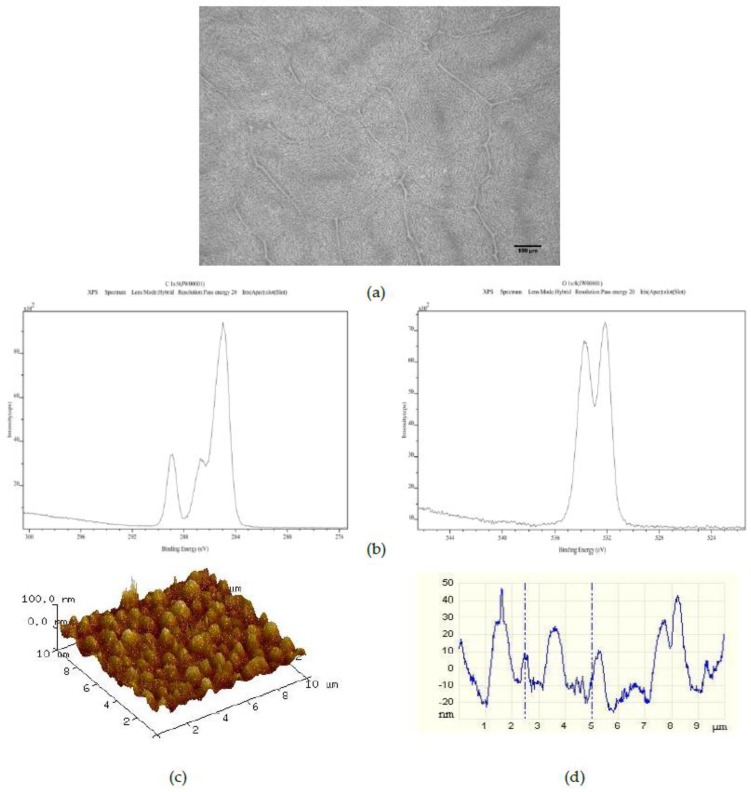
Characterisation of 25PS/75PMMA + 1500 films by (**a**) optical microscopy (×20); (**b**) C1s (**right**) and O1s (**left**) XPS spectra; (**c**) AFM 3D plots and (**d**) AFM line profile.

The effect of adding 1500 50 μm PS microspheres to the 25PS/75PMMA polymer demixed solution is shown in Figure 4. Once more, the optical image (Figure 4a) shows the striations formed by the PS particles. However, these are now somewhat more randomly distributed than was the case for the 25PS/75PMMA + 750 demixed sample. The C1s and O1s XPS spectra (Figure 4b) indicate that the chemistry of the surface is still PMMA. The AFM image and line profile (Figure 4c,d) here show pronounced recurring features in the 20 nm to 50 nm height range with FWHM values of approximately 500 nm.

**Figure 5:**
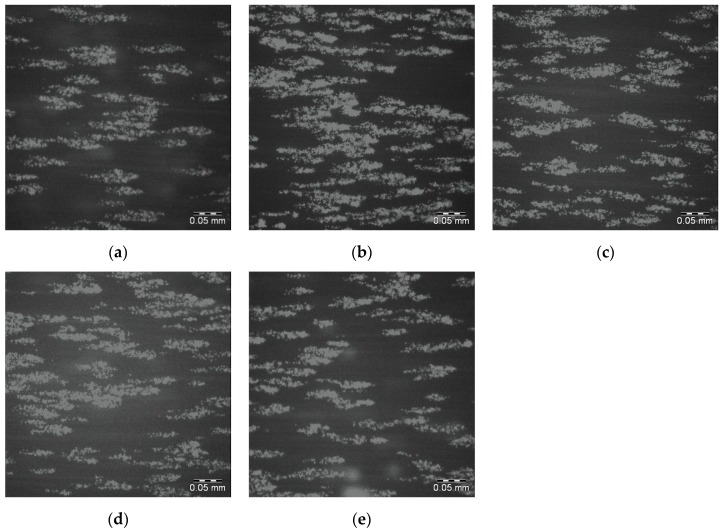
Fluoresence micrographs of whole blood DIOC_6_ labelled platelets adhered to the surface of each of 5 replicates (**a**–**e**) of the 25PS/75PMMA demixed spin coated surface. Scale bar 0.05 mm.

The whole blood, DIOC_6_ labelled platelet interactions with the 25PS/75PMMA spin coated films on glass slides has been determined by fluorescence microscopy. Images from within the DPFA microfluidic flow path were acquired over a total exposure period of 4 min. Figure 5a–e shows the final image (120/120) in the sequence for each of 5 replicates of this surface with significant numbers of adhered platelets seen in all cases. The average percentage surface coverage of platelets (pixels associated with adhered platelets divided by the total number of pixels) is reported in Table 3.

**Figure 6:**
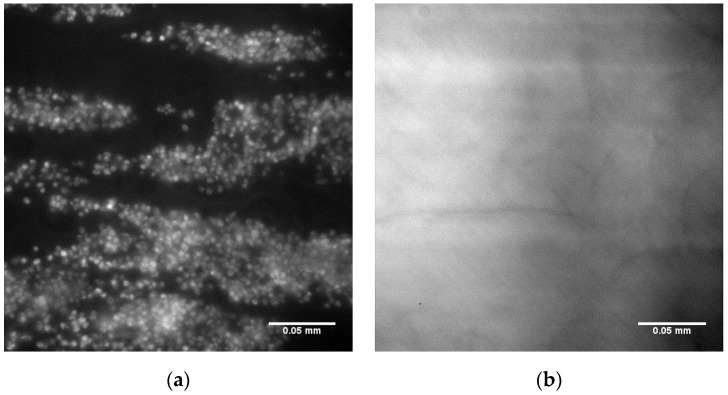

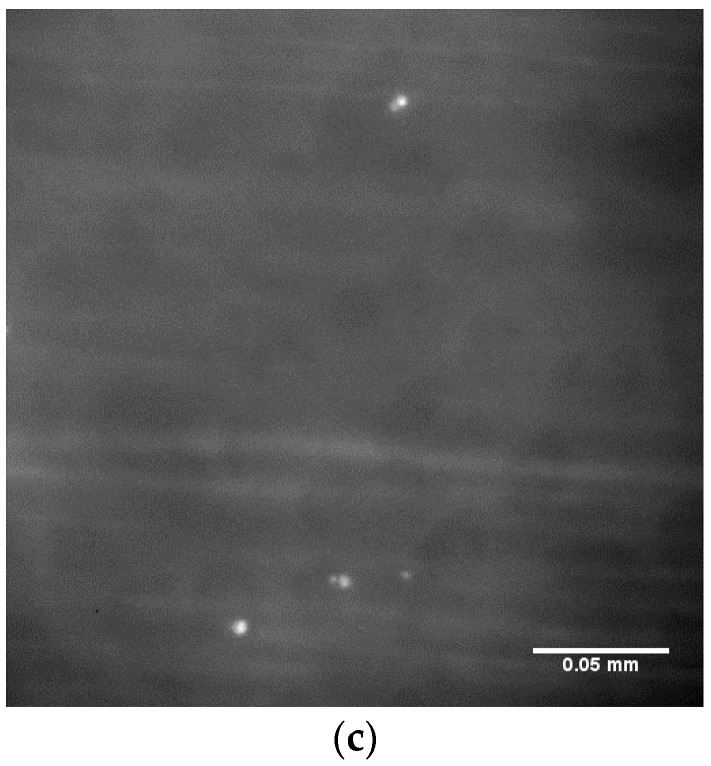
Fluoresence micrographs of 25PS/75PMMA demixed surface (**a**) with whole blood DIOC_6_ labelled platelets adhered to the surface; (**b**) after addition of AK2 platelet antibody and (**c**) after inclusion of 5D2 vWF antibody. Scale bar 0.05 mm.

To confirm that the interaction of the platelets with this polymer demixed spin coated surface is specifically due to the presence of uncoiled vWF, inhibitory antibodies were added to the blood samples prior to flow over the substrate in the parallel plate flow chamber. Specifically, the AK2 antibody was used to inhibit the GP1b platelet receptor and the 5D2 antibody used to inhibit vWF binding sites. As shown in Figure 6, both antibodies were found to completely inhibit platelet adhesion on this surface.

**Figure 7:**
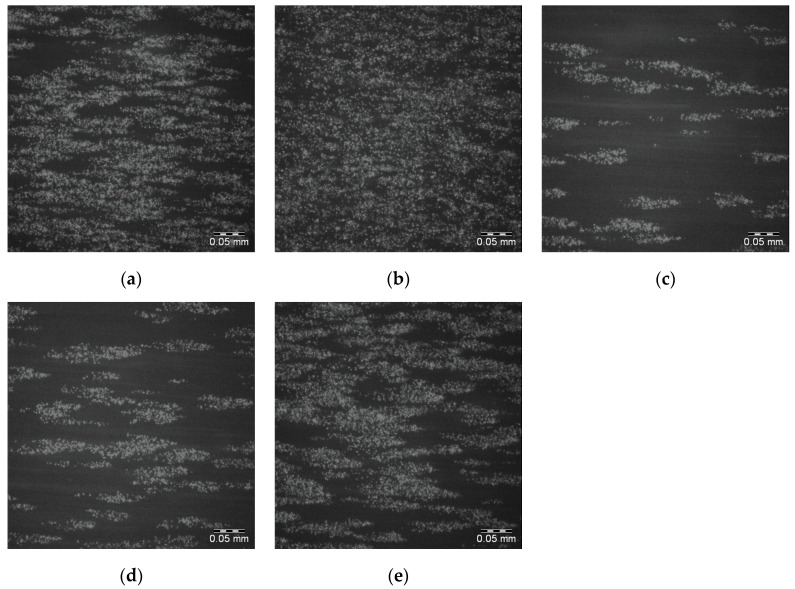
Fluoresence micrographs of whole blood DIOC_6_ labelled platelets adhered to the surface of each of 5 replicates (**a**–**e**) of 25PS/75PMMA + 750 polymer demixed surface. Scale bar 0.05 mm.

The effects of adding 750 50 μm PS microspheres to the spin coating solution of 25PS/75PMMA in chloroform on the adherence of whole blood DIOC_6_ labelled platelets is shown in Figure 7a–e. The attendant percentage surface coverage of the platelets to this surface are provided in Table 3. Images were not acquired for the 25PS/PMMA75 + 375 surface as its chemical and topographical properties were too similar to those of the standard 25PS/75PMMA sample. In general, for the surfaces in Figure 7a,b,e, platelet coverage is greater and more coherent. However, Figure 7c,d show behaviour that is broadly similar to that observed for the standard (25PS/75PMMA) samples.

**Figure 8:**
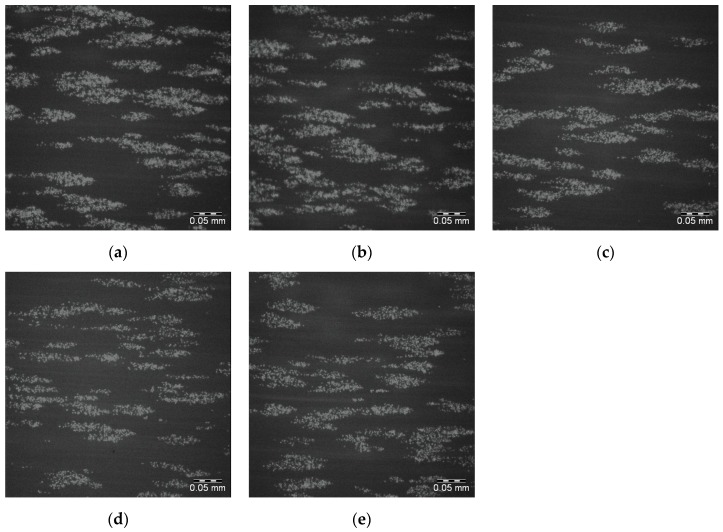
Fluoresence micrographs of whole blood DIOC_6_ labelled platelets adhered to the surface of each of 5 replicates (**a**–**e**) of 25PS/75PMMA + 1500 polymer demixed surface. Scale bar 0.05 mm.

The corresponding images for the 25PS/75PMMA + 1500 demixed surfaces are shown in Figure 8a–e with the surface coverage data again provided in Table 3. The sample to sample platelet coverage in these images is similar to the behaviour recorded for the 25PS/75PMMA standard surface.

In order to determine if the variation in the platelet surface coverage observed for the 25PS/75PMMA + 750 demixed surfaces is a consequence of inconsistency in the sample to sample surface features, additional AFM analysis was carried out at 3 different points on each of the 5 samples along each flow path. These data (not shown) indicated that the respective *R*_a_ and *R*_q_ values were very similar for all areas measured in each of five samples with and without the addition of 750 and 1500 50 μm PS microspheres. In addition, water contact angle measurements were made (CAM200, KSV Instruments, Helsinki, Finland) operating with a 5 μL droplet on each of the main samples to see if there was variation in the attendant wettability with values as follows: 25PS/75PMMA = 65.5° ± 1.3°; 25PS/75PMMA + 750 = 73.5° ± 2° and 25PS/75PMMA + 1500 = 73.7° ± 2.3°.

## 4. Discussion

Direct observation of the coatings created by spin coating the solution of 25PS/75PMMA onto glass slides shows a distribution of solidified PS particles embedded in PMMA with rounded features and long striations that radiate in various directions. Whereas, this type of optical observation has not previously been made for PS/PMMA demixed systems, it has been reported that films created using a blend of 95% Polyethyleneimine (PEI) and 5% Polycaprolactone (PCL) in a dichloromethane (DCM) have two distinct morphologies that broadly agree with the observations made here [21].

The surface chemistry of the 25PS/75PMMA spin coated surfaces was found to be only that of PMMA with no evidence of PS identified at the detection level of the XPS method employed (c.a. 0.1 %At. Conc.). Previous studies of the surface chemistry and nanotopography of such polymer demixed thin films with a high concentration of PMMA indicates the formation of a nano-island topography, whereas when PS was more dominant (≥50%) a nano-pit arrangement was observed [19,20,21]. The influence of the concentration and composition of the PS/PMMA mixture on the morphology and overall configuration of the resulting surface has also been reported previously [22,23,24]. A consideration of the thermodynamic conditions at play when the polymer demixing process that occurs when a spin coated surface is formed from a mixture of PS and PMMA, suggests a significant factor is minimisation of the surface tension [25]. The expectation would then be that PS would dominate in the subsequent formation of the top surface region of the film. The fact that this is not the case can be explained to be as a result of using chloroform in the casting solution. The use of this highly volatile solvent, means that the polymers do not have enough time to reach thermodynamic equilibrium in solution, resulting in the PMMA enrichment at the surface due to a combination of several factors—the substrate type, the film thickness and the polymer chain structure [26,27]. The data presented here confirm that the resultant surface region is caused by the highly soluble PMMA in chloroform flowing over the mostly insoluble PS particles during the spin coating process such that when the solvent evaporates, the PMMA completely encases the PS in a way that produces the resulting micro-/nano-structure [28,29,30]. The surface topography, as measured from the AFM images and associated line profiles here, comprised a mixture of micron to nano-scale features in the range from 4 μm to 100 nm. These data are consistent with other results for features created by spin coating the polymer demixed PS/PMMA systems [19,22].

Importantly, the surfaces produced here have been shown to be capable of entrapping vWF from flowing blood in a manner that allows for subsequent platelet interactions to occur thereon. Moreover, the addition of inhibitory antibodies to the blood samples that block key binding sites confirm that the surface is also capable of presenting the uncoiled form of vWF to the platelets [31]. Hence, these surfaces clearly have potential for use as substrates for the direct capture of autologous vWF in the DPFA measurements of interest. The surface coverage values obtained and the associated adhered platelet distributions correlate with the irregularities observed in the attendant topography. As such, in this form, the 25PS/75PMMA surfaces are not capable of providing the reproducibility necessary to ensure accurate clinical DPFA test data. Minelli et al. treated similar PS/PMMA spin coated surfaces with cyclohexane, which after exposure to whole blood showed a higher density of platelets firmly adhered to larger surface features but the overall coverage was still sporadic [19,20].

The introduction of various numbers of 50 μm PS microspheres into the 25PS/75PMMA spin coating solution was used here as a means to enhance the size and uniformity of the surface topographical features. The principle of operation here is similar to that employed by Ton-That et al., whereby fluid mechanics plays a major role in determining the surface topography that is formed in such systems [28,29]. Although the inclusion of 375 microspheres per mL in the casting solution has little or no effect, increasing this number to 750 per mL does have a positive effect on the both the regularity and promotes average surface features at the 40 to 50 nm height scale with FWHM of approximately 1 μm. Doubling the number of microspheres in the cast solution again to 1500 per mL produces coatings with surface features similar to the standard coatings. In all cases, the surface chemistry is again determined to be that of PMMA only. The PS microsphere were added as a way of disturbing the PS within PMMA more evenly and therefore modifying the attendant nanotopography. AFM studies show that the feature size height decreases after addition of 750 50 μm PS microspheres and also has a much more consistent size distribution in 3 of 5 samples. Addition of 1500 50 μm PS microspheres results in a surface topography with reduced feature heights (20 nm to 50 nm) and FWHM (500 nm) compared to the 750 samples but with a less consistent distribution.

The various spin coated surfaces produced from 25PS/75PMMA solutions with 50 μm PS bead are again all shown to be capable of vWF entrapment with the protein in the uncoiled state as determined by subsequent adherence of platelets thereon. The surfaces created with 375 PS microspheres shows similar surface coverage to that seen for samples created with standard cast solutions. However, in the case of the surfaces with the topography resulting from the presence of 750 50 μm PS microspheres, the surface coverage is greater and the associated distribution is more even across the whole region imaged. Some sample to sample variation in platelet surface coverage was observed for this surface type. Additional checks indicated that the *R*_a_ and *R*_q_ values were very similar for all three areas measured in each of five samples. Water contact angle measurements indicate a small change in surface wettability between the 25PS/75PMMA and 25PS/75PMMA + 750 samples. Whereas, it is accepted that surface wettability is mainly due to chemistry, surface roughness does play a role. Since the extensive use of XPS analysis indicates that the surface chemistry of the PMMA does not change with the introduction of the PS microspheres the wettability does. Hence, these data suggest that for these surfaces, the change in topography does influence the slight change in wettability observed. Overall, the surface features produced are consistent in the context of provision of a surface capable of entrapping autologous vWF for use in the DPFA device, however, its reproducibility still needs improvement. By comparison, the 25PS/75PMMA + 1500 samples show a platelet response that is similar to the standard 25PS/75PMMA surface (and the 25PS/75PMMA + 375 samples). This suggests that not only is the topographical feature size important, but so also is the regularity of its distribution across the region of interest.

## 5. Conclusions

Surfaces produced by spin coating a 25PS/75PMMA demixed solution in chloroform onto glass slides have been shown to have topographical features that are capable of the direct entrapment of autologous von Willebrand Factor (vWF) in a form that can induce subsequent platelet interactions within the same blood sample. The nature of the vWF platelet interaction has been established as being physiologically relevant by the use of antibodies to block binding sites. As such, these substrates show promise for use within a DPFA test device that combines a top plate with in/out fluid ports with the polymer demixed coated slide as the bottom part of a microfluidic flow chamber. However, reproducibility of this autologous vWF substrate surface is a critical factor for its clinical utility. Inclusion of varying numbers of 50 μm PS microspheres in the 25PS/P75PMMA demixed system has been shown to offer a means of regulate the topographical features without changing the attendant surface chemistry. In particular, the addition of 750 microspheres produces highly reproducible surface features (50 μm high; 1 μm FWHM) that entraps vWF in a manner that provides for an increased and more coherent surface coverage across the test area.

## Figures and Tables

**Table 1:** XPS quantitative data (% atomic concentration (*n* = 3)) for O1s and C1s contributions to 25PS/75PMMA demixed spin coated surfaces with and without addition of 375, 750 and 1500 50 μm PS microspheres. Data for a spin coated 100% PMMA film is included for comparison.

Sample Type	Sample Number	%At. Conc. Carbon (C1s)	%At. Conc. Oxygen (O1s)	Average %At. Conc. Carbon (C1s)	Average %At. Conc. Oxygen (O1s)
100% PMMA	1	80.53	19.47	80.23 ± 0.37	19.77 ± 0.37
	2	80.35	19.65		
	3	79.82	20.18		
25PS/75PMMA	1	76.56	23.44	78.41 ± 1.60	21.59 ± 1.60
	2	79.23	20.77		
	3	79.43	20.57		
25PS/75PMMA + 375	1	79.65	20.35	77.68 ± 1.87	22.32 ± 1.87
	2	77.78	22.52		
	3	75.92	24.08		
25PS/75PMMA + 750	1	80.26	19.74	78.45 ± 2.96	21.55 ± 2.96
	2	80.06	19.94		
	3	75.04	24.96		
25PS/75PMMA + 1500	1	76.61	23.39	76.45 ± 0.20	23.55 ± 0.20
	2	76.50	23.50		
	3	76.23	23.77		

**Table 2:** Mean (*n* = 3) surface roughness (*R*_a_) and root mean square roughness (*R*_q_) values calculated from AFM images acquired for 25PS/75PMMA demixed spin coated surfaces with and without addition of 375, 750 and 1500 50 μm PS microspheres per mL. Data for a spin coated 100% PMMA film is included for comparison.

Substrate	Average *R*_a_ (nm)	St Dev (*R*_a_)	Average *R*_q_ (nm)	St Dev (*R*_q_)
100% PMMA	0.32	0.01	0.53	0.02
25PS/75PMMA	12.93	4.06	15.85	4.72
25PS/75PMMA + 375	15.20	4.98	18.57	5.74
25PS/75PMMA + 750	15.72	5.04	19.14	5.40
25PS/75PMMA + 1500	11.02	3.90	15.08	1.52

**Table 3:** Average % surface coverage (*n* = 5) and standard deviation values for 25PS/75PMMA spin coated surfaces with and without addition of 750 and 1500 50 μm PS microspheres.

Solution	Sample #	% Coverage	Average	Standard Deviation
25PS/75PMMA	1	7.94	13.89	6.60
	2	24.64		
	3	14.85		
	4	12.70		
	5	9.30		
25PS/75PMMA + 750	1	32.50	19.08	12.59
	2	28.65		
	3	3.65		
	4	8.37		
	5	22.22		
25PS/75PMMA + 1500	1	16.76	10.88	4.11
	2	13.00		
	3	10.34		
	4	6.82		
	5	7.47		
